# The cross technique for the positioning of Kerboull plate in acetabular reconstruction surgery

**DOI:** 10.1051/sicotj/2018012

**Published:** 2018-06-04

**Authors:** Chahine Assi, Jacques Caton, Thierry Aslanian, Camille Samaha, Kaissar Yammine

**Affiliations:** 1 Lebanese American University Medical Center-Rizk Hospital, Department of Orthopedics, Beirut Lebanon; 2 Institut de Chirurgie Orthopédique, Lyon France; 3 Groupe Lépine, Genay France

**Keywords:** Total hip arthroplasty, revision hip, kerboull plate, acetabular reconstruction

## Abstract

Acetabular reconstruction aims at filling bony defects and positioning the acetabular component in its anatomical place. To this, the use of Kerboull plate would give an automatic optimal positioning while reconstructing the acetabular cavity. We developed a technique, the cross technique, that approaches acetabular reconstruction in a systematic way. It consists of placing the KP following its cross design, in reference to a vertical plane (hook, center, palette) and a horizontal plane (horizontal flanges). The cross technique has three steps; the preparation of the acetabular cavity, the cross technique itself and cementation. We recommend a downsizing of the plate in relation to the size of the acetabular cavity in addition to another downsizing of the acetabular implant in relation to the plate size whenever a dual mobility cup is used.

## Introduction

Since its first description by Marcel Kerboull [1], the Kerboull acetabular reinforcement plate device or Kerboull plate (KP), demonstrated excellent mid- and long-term outcomes in relation to the survivorship of the acetabular implants in total hip arthroplasty (THA) [1–4]. As it is known, the aim of a THA, whether primary or revision, is to restore the anatomy in order to obtain the original rotational center of the hip. To this, the KP serves as a frame guide for acetabular reconstruction and cup positioning. Recently, many reports of its use in THA with dual mobility cups (DMC) were published in the literature with excellent mid-term survivorship results [5,6]. The technique of positioning the KP has been subject to few publications with some variants. Since no standardization could be found, we elaborated a new technique simple to reproduce while being adapted to the different surgical approaches and possible acetabular defect situations. The primary aim of this paper is to describe the technique being used, that we named the cross technique, that includes the positioning of the KP in two planes. The indications of use of the KP and the classifications of the acetabular defects will not be discussed in this paper.

## The surgical technique

We used a replicate of the original Kerboull plate design: a proximal palette bearing four holes for screw fixation, vertical flanges crossed by horizontal flanges, and a distal hook. The only difference with the original design is that the central hole of the cross can bear a handle to hold the plate, referred to as the Caton handle (Groupe Lepine, Genay, France).

The cross technique is a practical and systematic approach for the application of the KP that helps in reconstructing the acetabulum in primary and revision THA. This technique has been used and developed in our department since 12 years ago when we started using DMC. The surgical description includes the cross technique itself along with 2 steps; the preparation of the acetabular cavity preceding the cross technique itself and the cementing method following it.

It consists of placing the KP in reference to 2 planes, the vertical and horizontal planes, after preparation of the acetabular cavity. The cross technique steps are in the following order; (1) preparation of the acetabular cavity, (2) positioning of the elements in the vertical plane (the hook, the curved vertical KP center and then the palette), (3) positioning of the elements in the horizontal plane (the anterior and posterior flanges), and (4) cementing.

### Preparation of the acetabular cavity

The acetabular cavity is widely exposed after removing the osteophytes. When present, the annular ligament is conserved to assure the primary stability of the hook (see next step).

In case of a revision surgery and after removing the acetabular implant, soft reaming of the acetabular cavity at a slow speed is performed to remove the fibrotic and adherent fragments without weakening the remaining bone stock. That would result in a harmonious and spherical acetabular cavity to reconstruct. Only the peripheral bone is subject to this careful reaming; fibrosis and/or cement on the medial wall are removed carefully with big curettes and rongeurs to avoid breaching the medial wall. The use of small curettes is avoided because of the increased likelihood of breaching the bone.

### The vertical plane

Three elements of the vertical plane are checked in the following order; the hook, the center of the KP and the horizontal palette.

#### The distal element: position of the hook

The fibrotic tissue and the annular ligament are maintained on the inferior edge of the acetabulum when this edge is not destroyed. A long Mayo scissor placed on the superior border of the obturator foramen and taking contact with the bony floor is slid from proximal to distal in order to enter the space between the bone and the annular ligament. Using a long Cobb elevator having a width of no more than 1 cm, the entry opening is widened with gentle wrist movements enough to accept the hook. In this, the primary stability of the hook is assured by the ligament and the fibrotic tissue on the residual bone. The opening should be made in the middle of the acetabular edge. In case of anterior defects, mild posterior translation of the opening is preferred where the ischiatic bone is usually well preserved.

If the lower margin of the acetabulum is disrupted, a graft is placed in the hook and using the Caton handle on the plate, the hook is brought in between the anterior and posterior horns of the inferior acetabular edge.

The size of the KP could be estimated either by radiological measurements of the contralateral hip when possible, by multiple trials, or by the size of the removed acetabular implant. The size of the KP is usually 1 or 2 sizes smaller than the last reamer used.

#### The central element: vertical position of the center of the KP

**Figure 1:**
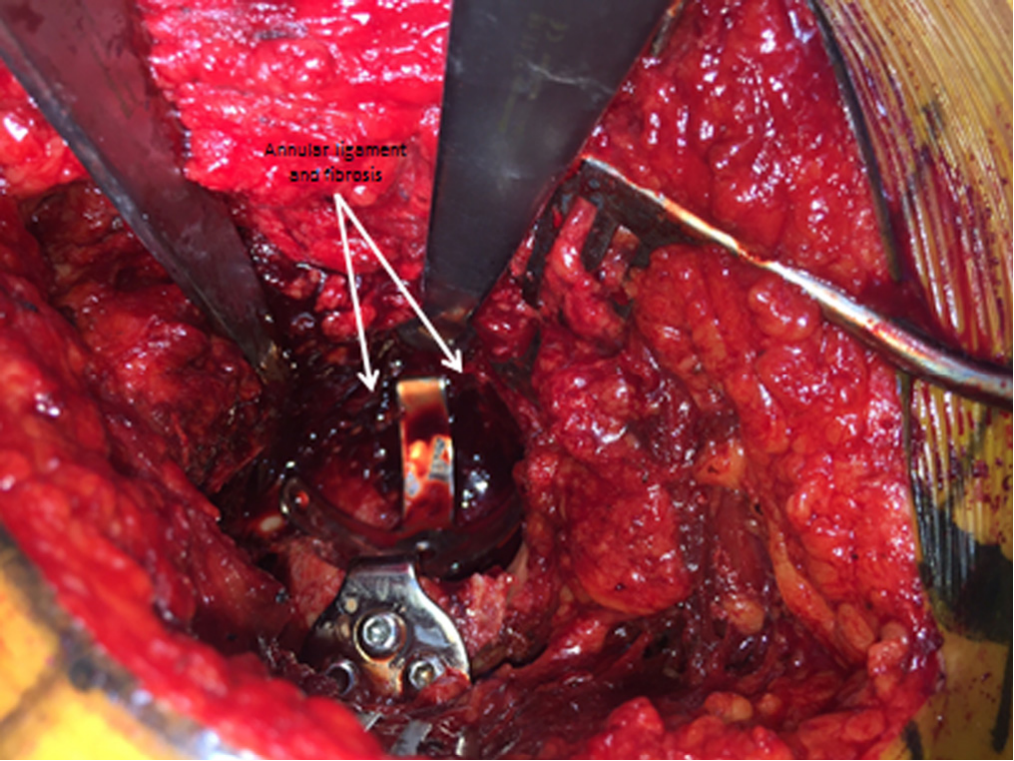
Annular ligament and fibrotic tissue are not removed.

**Figure 2:**
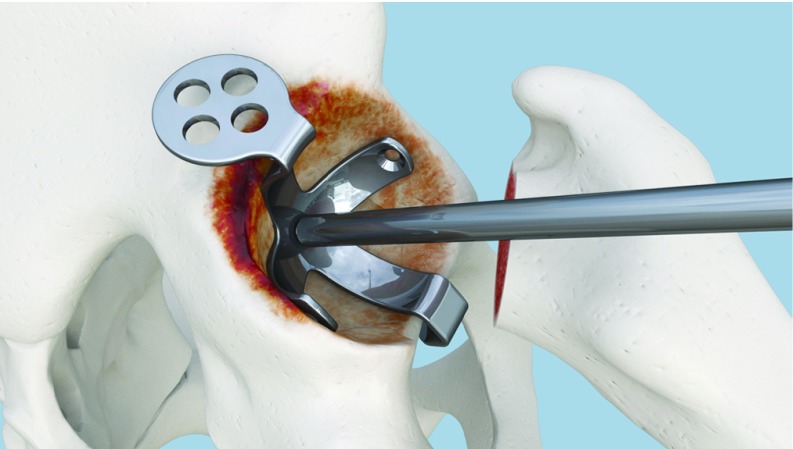
Alignment between cavity center and plate center using Caton handle.

When the medial wall is preserved, the center of the KP is positioned following the center of the medial wall. In case of revision surgery, this could be sometimes difficult when parts of the medial wall are disrupted. The Caton handle used to hold the KP trial chosen is very helpful to estimate the alignment between the center of the cavity and the center of the KP (Figures 1 and 2). Using this handle, the trial KP is impacted medially and adjusted to obtain an optimal alignment of both centers.

In case of a large defect of the medial wall (Protrusio acetabuli), a reconstruction with a single graft is preferred over multiple bony fragments or cancellous graft. Again, trials are conducted to narrow down the number of best matched sizes of the KP.

#### The proximal element: position of the horizontal palette

**Figure 3:**
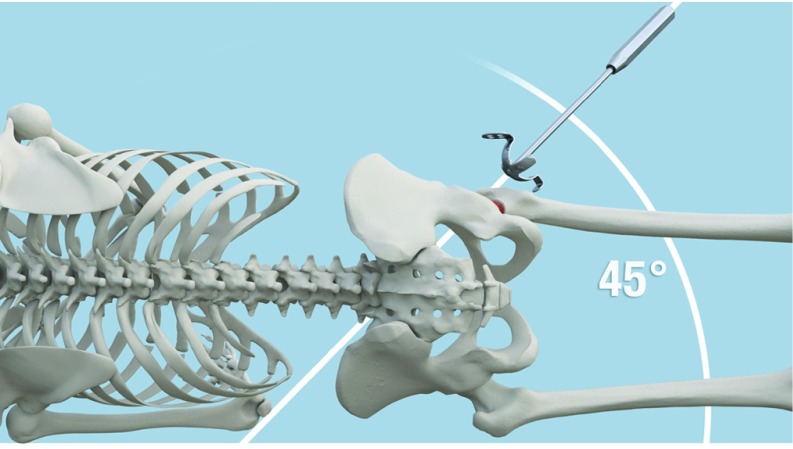
Inclination of 45 degrees of the plate.

**Figure 4:**
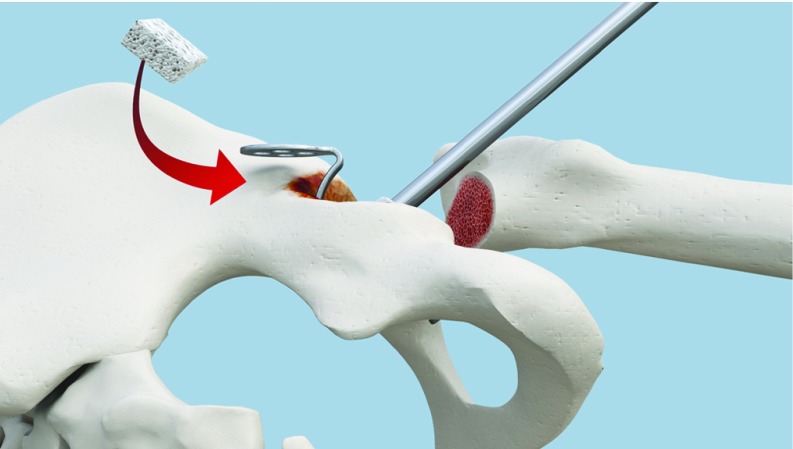
The gap is filled with a graft. Lateral view.

**Figure 5:**
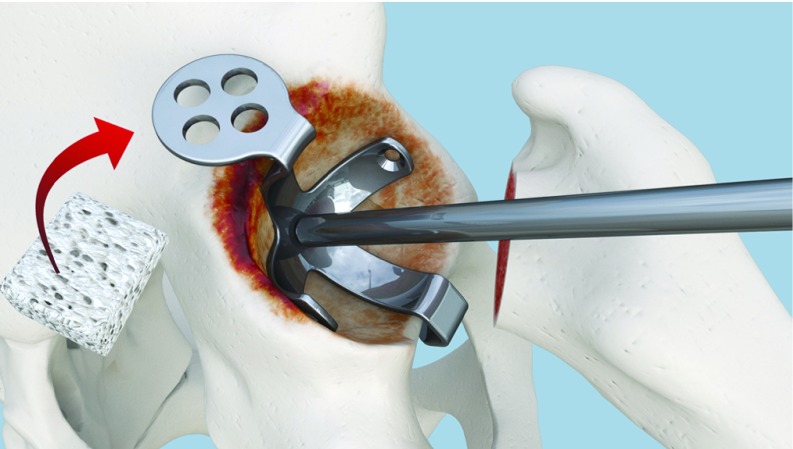
The gap is filled with a graft. Upper view.

**Figure 6:**
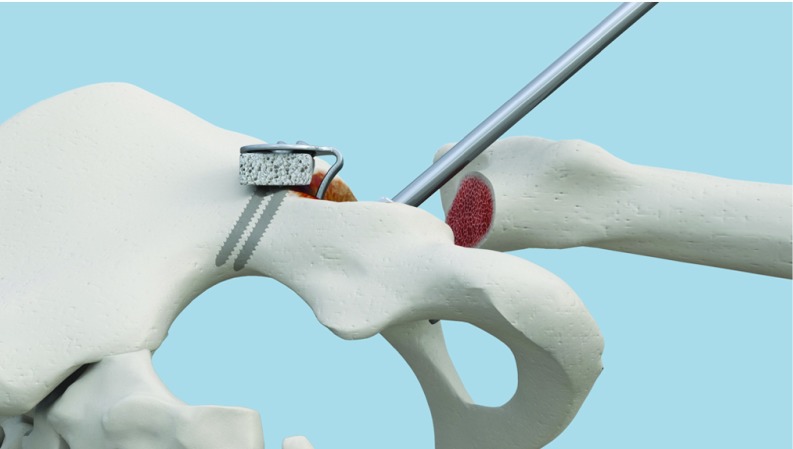
The superior graft in place.

To obtain an inclination of 45 degrees of the KP, the hook should be adjusted in order to position the horizontal palette parallel to two references: the floor and the surgical table (Figure 3). The KP is designed in a manner that, when the palette is horizontal, it automatically gives an inclination of 45 degrees with regard to the KP. Again, the Caton handle is designed to facilitate this perfect positioning by maintaining the palette horizontally. Bending of the palette is prohibited for many reasons that will be further detailed in the discussion. Thus and even in cases of minor superior defects, it is recommended to fill the gap with a graft rather than bending the palette (Figures 4 and 5). The bone should be adapted to the palette and not the opposite (Figure 6).

**Figure 7:**
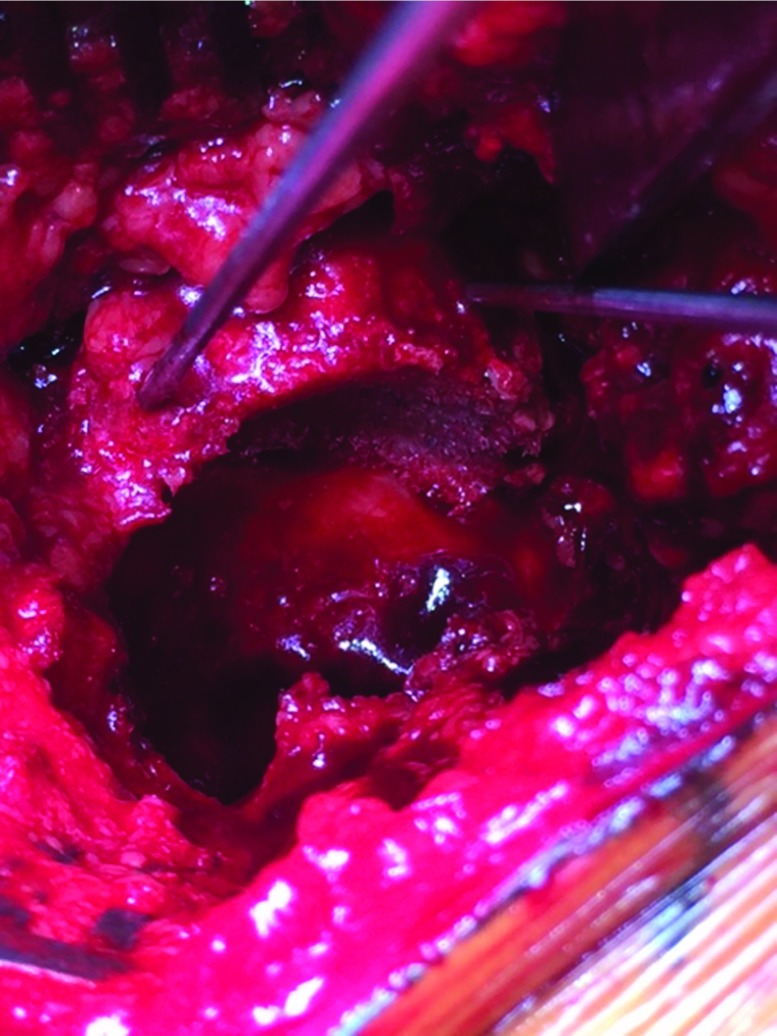
After cavity reaming with the superior graft fixed by 2 temporary wires.

Usually superior defects are best managed by a single bone graft. After fixation to the iliac bone by temporary convergent 2 k-wires (Figure 7), the graft is flattened on its superior aspect to accommodate the horizontal palette. Its inferior surface is then gently reamed to get a harmonious concave shape and a continuous the remaining of the acetabular cavity.

### The horizontal plane

Once the vertical and central steps are achieved, the horizontal plane consists in positioning the anterior and posterior flanges of the KP.

This step is easier to accomplish when both the anterior and posterior walls are preserved. In case of bony wall defect, three situations could be faced. When the defect is limited to the anterior wall, the preserved posterior wall is used to match the posterior flange for KP size estimation. In the event of an isolated posterior wall defect, the surgeon can rely on the anterior wall for matching and sizing. When both walls are involved, reconstruction using big fragments is first performed before a sizing trial is conducted. In the latter case, any existing acetabular bone reference can be used as a circle arc that will be matched and completed with the trial KP. In all cases, the horizontal flanges should not extrude outside the reconstructed bone cavity (Figure 2).

At this step, the final KP size is chosen and by means of Caton handle, the last adjustment to obtain the planned positioning of the KP in the horizontal plane can be fine-tuned.

Once all fragments are in place, morcellized cancellous bone is packed between the interstices. Reconstruction of the defects of both walls is guided by the amount of gap found between the defects and the KP held in its presumed final position. When possible, the embedded grafts are secured to the remaining bone using screws. At this stage defects of the walls can be filled after fixation of the KP as well.

## Fixation of the KP

**Figure 8:**
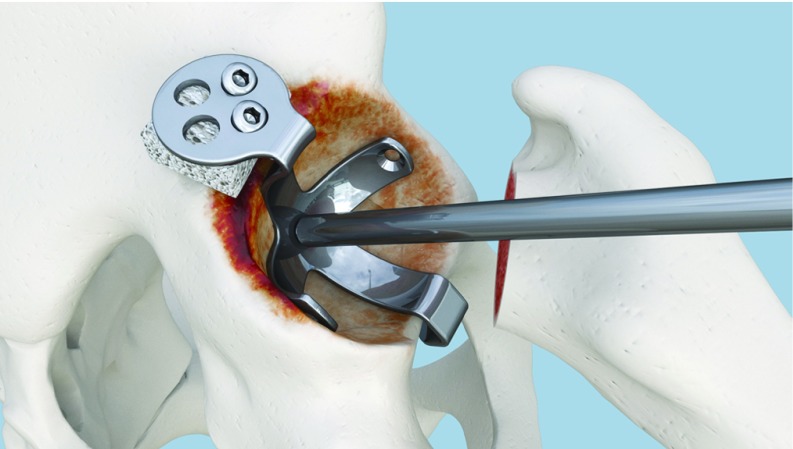
Fixation of the palette to the graft and iliac bone.

**Figure 9:**
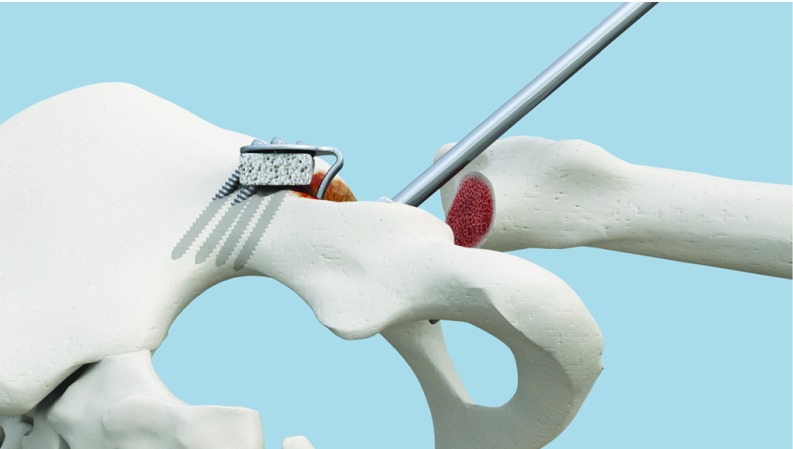
Fixation completed. Lateral view.

**Figure 10:**
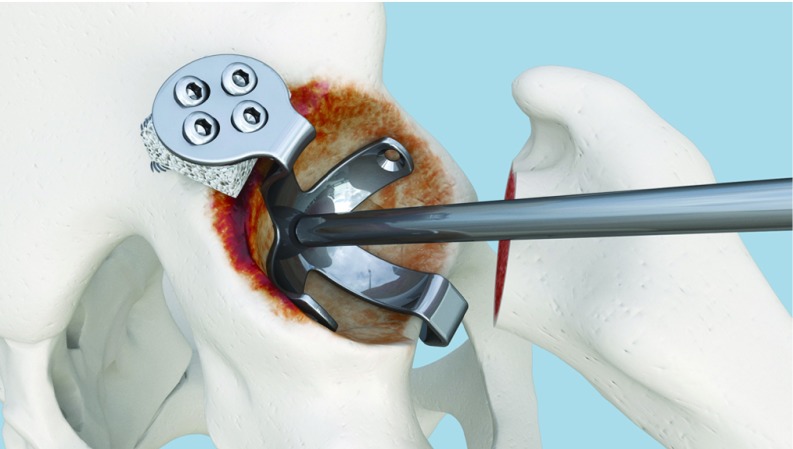
Fixation completed. Upper view.

Before fixing the KP to the bone, no space should exist between the superior roof and the horizontal palette, any defect is filled with bone graft. While implementing a lateral to medial pressure on the KP, the inferior screw is inserted first but not tightened followed by the lateral screws. All 3 screws are then tightened while keeping manual pressure on the KP. In this, we avoid any displacement of the KP via a progressive and simultaneous tightening of all 3 screws. The superior vertical screw is inserted last and tightened to secure the construct (Figures 8–10). The technique of drilling aims at reaching the inner cortex without penetrating it first to avoid injury of the neighboring intra-pelvic vessels. Once the drill reaches the outer cortical layer, gentle tapping with the drill is completed before perforating the inner cortical layer.

## Cementing step

**Figure 11:**
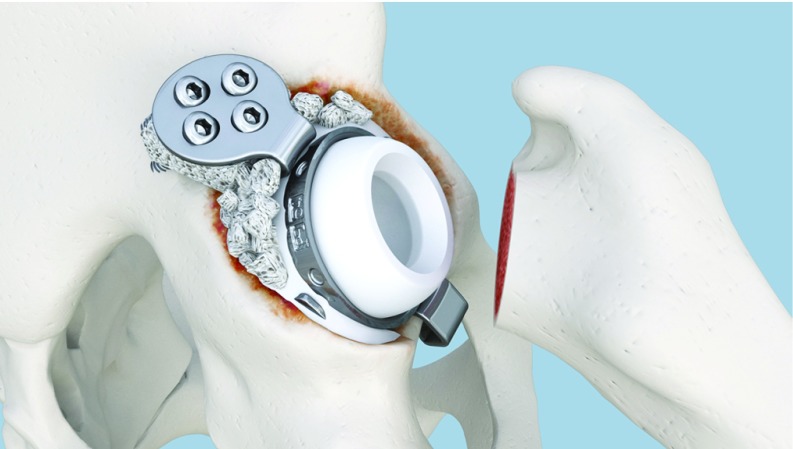
Cemented DMC in place.

After mixing, the cement is divided into two portions. One will be used to tap and pack the cortical and cancellous bone graft used to reconstruct the acetabulum; that will avoid cement leakage and seal the different packed grafts. Immediately afterwards, the backside of the implant is covered with the other portion of the cement and impacted in the KP. In case of use of a dual mobility cup (DMC), we choose to downsize the cup by 2 sizes in relation to the KP size (Figure 11). Eventually a gap could persist between the superior graft and the KP; in such case the space is then filled with cement.

**Figure 12:**
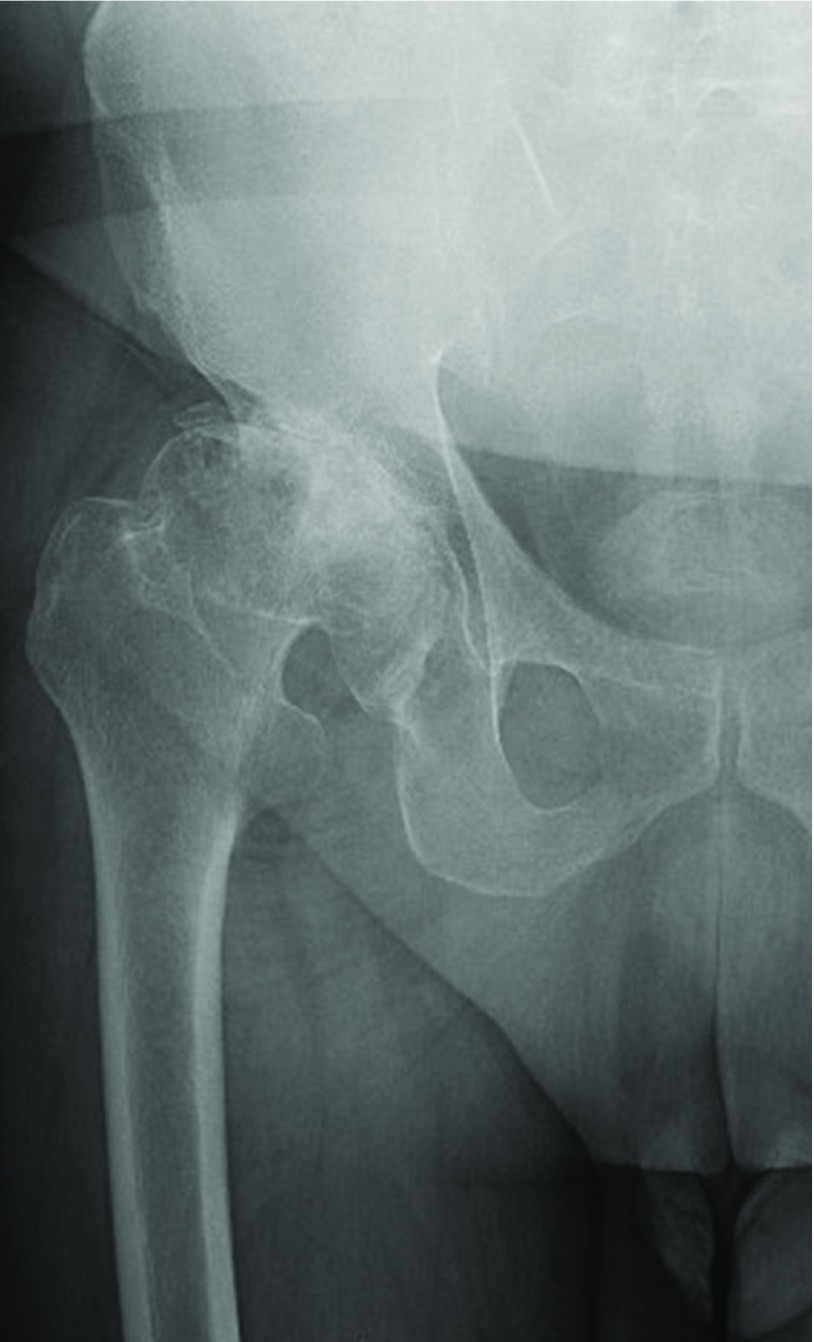
Pre-oprative X-ray.

**Figure 13:**
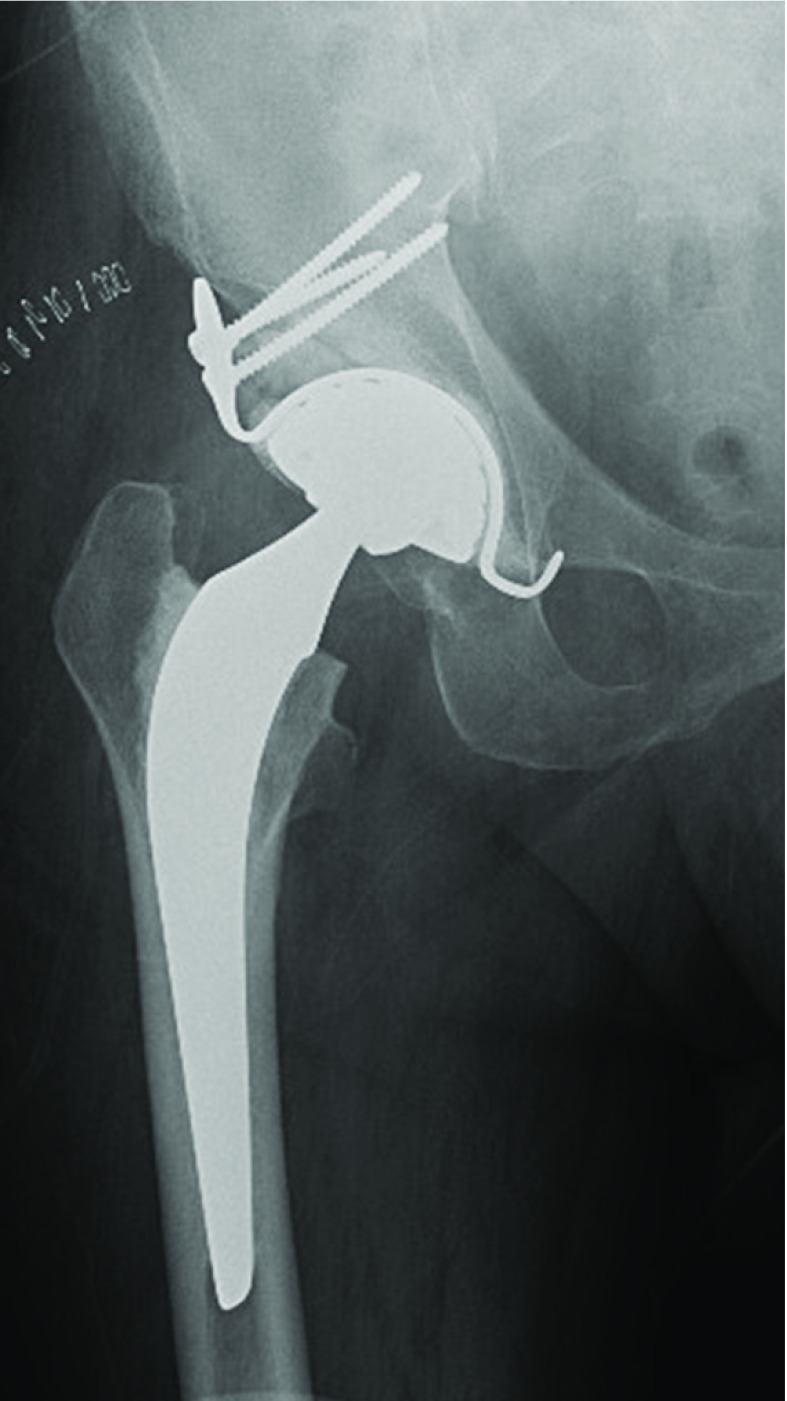
Post-operative X-ray.

A clinical case of THA using a DMC and a KP is shown in Figures 12 and 13.

## Discussion

In this paper, we described a systematic technique of KP placement. The cross technique naturally follows the cross design of the KP in its 2 planes. After preparation of the acetabular cavity, the three elements of the vertical plane are adjusted first before proceeding to the fine-tuning of the single element of the horizontal plane.

Our technique attempts to enhance the easiness of the excellent original method of acetabular reconstruction via the Kerboull plate. It includes some technical and instrumental differences. For instance, soft reaming is always used for cavity preparation. We believe that reaming could be better than the use of curved curettes in removing the peripheral fibrotic tissue mainly for 2 reasons; first, the reamer follows the sphericity of a preserved cavity and helps in creating a spherical cavity when bone defect is present, and it is less likely to accidentally breach of the bony wall.

It is known that a trochanterotomy as it is systematically performed by Kerboull and Kerboull [1], provide a wider surgical field exposure in comparison to other approaches. Since a trochanterotomy was never performed, the Caton handle was found to compensate a potential field view limitation when the posterolateral approach is used. Instead of maintaining a direct continuous pressure on the hook during the whole procedure to avoid it expels as it is described in the original technique, the handle would maintain the hook in its place with no need to continuously monitor its location by keeping retractors in the inferior field.

We believe that the preservation of the annular ligament and the fibrotic tissue is crucial for the primary stability of the hook, and consequently of the KP. Such distal anchorage would help in properly positioning the center of the KP and the palette later on. It is noteworthy to stress on the importance of limiting the width of the opening to that of the hook size; the distal end should forcefully enter this opening created by the Cobb elevator. This will insure hook stability before moving on to the next step. In case of a disrupted annular ligament and/or a defect of the inferior bony edge of the acetabulum, we find that incorporating the graft in the hook in a single step is easier than placing a graft in the inferior defect and trying to fit the hook inside. Because of the difficulty in obtaining the exact size, the graft is often unstable when placed between the two horns of the inferior acetabulum. Owing to the shape of the distal hook, a graft of a proper thickness could fit and held spontaneously in place. The KP including the inferior graft could be brought easily to the acetabular edge by means of the Caton handle.

In regard to the horizontal palette, instructions of the original technique should be followed and the palette should not be bent. In case of a minor defect, many surgeons could be tempted to bend the palette downward to obtain bony contact. There are three sound reasons to avoid bending the palette; the design is made in such that the bone should adapt to the KP and not the opposite, the distal hook often expels when applying a vertical pressure on the palette, and last there is a risk of palette weakening at the angle where it connects with the vertical branch. Some authors reported the use of variants of the original KP to get a higher placement of the implant [7–9].

The Caton handle was found to be extremely helpful in positioning and maintaining the KP in every step of the technique. In addition to its role in the first step, the handle assists in aligning the center of the KP with the center of the cavity in the second step. In the third step, it helps maintaining the palette parallel to the surgical table/floor, assuring a stable inclination of 45° of the KP throughout the procedure.

While autograft or allograft using femoral heads are used to reconstruct large superior roof or medial wall defects, small chips are used to fill the gaps between large fragment grafts [10]. Morselised fresh-frozen allograft has been considered as the standard graft in such case [11]. Irradiated allograft show good results in a medium and long-term follow-up [12] while freeze-dried irradiated allograft treated chemically with lower allograft-related infection or illness, were used successfully in acetabular reconstruction [10].

We usually check and control two downsizings; one related to the KP and the other related to the use of a DMC. Downsizing the KP in relation to the bony cavity permits to obtain a better centering of the KP closer to the native center of the acetabular cavity, to avoid extrusion of the anterior and/or posterior flanges of the KP, and consequently diminishing the risk of impingement. The second downsizing is related to DMC implants; in opposition to the recommended cemented standard polyethylene cup size which should match that of the KP size, the acetabular DMC implant is chosen 2 sizes inferior to that of the KP. This medializes the DMC which could avoid impingement with the prosthetic femoral neck and keeps the cup fit inside the flanges. More, such downsizing lead to a higher cement thickness around the cup, and eventually a better fixation.

Lastly, our radiological criteria for an optimal positioning are as follows:

- the distal hook fully locked in the foramen obturator on both the antero-posterior (AP) and lateral views;
- a 45° of inclination of the plate on AP views;
- a vertical direction of the palette, perpendicular to a line joining both distal ischiatic tuberosities on AP views;
-  presence of a medial bony wall on both views;
- screws directed towards the very distal aspect of the sacro-iliac joint;
-  no gap should exist between the plate and cement.

## Conclusion

We described a simple-to-follow technique, the cross technique, for positioning of the Kerboull plate during acetabular reconstruction surgery. This cross technique follows the cross design of the KP. This technique is easy to reproduce, adapted to different surgical approaches and acetabular defects.

### Conflict of interest

Dr Assi and Dr Caton disclose financial support from Groupe Lépine. Dr Caton is the Editor-in-Chief of the SICOT-J. Mr Aslanian discloses non-specific support from Groupe Lépine. All other authors disclose no conflict of interest.
